# An Ominous Case of Uterine Rupture in an Unscarred Womb and Its Subsequent Management

**DOI:** 10.7759/cureus.57273

**Published:** 2024-03-30

**Authors:** Krishna Sailaja Sattiraju, Meenal Patvekar, Dipak Kolate

**Affiliations:** 1 Obstetrics and Gynaecology, Dr. Dy Patil Medical College, Hospital and Research Centre, Pune, IND

**Keywords:** multidisciplinary approach, multiparity, antenatal care, emergency obstetric hysterectomy, unscarred uterus, uterine rupture

## Abstract

Uterine rupture is a rare but critical obstetric complication that demands a swift and decisive intervention to ensure the well-being of the mother and fetus. We present a case report detailing the surgical management of a bizarre uterine rupture in a multigravida female with two previous vaginal deliveries and a previously unscarred uterus. This case highlights the challenges of treating and diagnosing, particularly in the Indian setting, an antenatally unregistered patient with rare obstetrical complications. Emphasizing the clinical challenges faced and the multidisciplinary approach employed for optimal outcomes, this report underscores the importance of a high degree of suspicion, early diagnosis, timely intervention, and comprehensive intraoperative and postoperative care in addressing this rare obstetric catastrophic event. This article's main focus is multicentric, aiming to showcase the obstacles to maintaining low maternal mortality and morbidity, the presence of inadequate awareness in society, and the importance of multimodal treatment and planning.

## Introduction

Uterine rupture in pregnancy is a rare yet serious obstetric complication that poses significant risks to both maternal and fetal health [1]. Despite its infrequency, the potentially catastrophic nature of uterine rupture necessitates prompt intervention and a thorough understanding of its etiology, risk factors, and clinical presentation.

The etiology of uterine rupture is complex and multifactorial, often arising from a combination of intrinsic and extrinsic factors. A primary contributor is the presence of uterine scars resulting from a history of uterine surgery, such as previous cesarean sections, myomectomy, a history of repeated dilatation and evacuation, uterine septum removal, and traumatic injuries. Additionally, factors such as grand-multiparity and malpresentation, obstructed labor, assisted vaginal delivery, and macrosomic babies contribute to increased vulnerability [2,3].

The compromised integrity of the uterine wall in scarred areas becomes a vulnerable site for rupture during subsequent pregnancies. In addition to surgical history, uterine anomalies, such as congenital malformations, may increase susceptibility to rupture [2]. Fetal factors, including macrosomia and malpresentation, further elevate the risk, and maternal factors, such as advanced age and the excessive use of uterotonic agents during labor, contribute to the overall predisposition.

Understanding the pathophysiology is paramount because uterine rupture jeopardizes fetal oxygenation and exposes the mother to life-threatening hemorrhage, shock, and disseminated intravascular coagulation [4]. Clinically, uterine rupture may manifest with sudden onset abdominal pain, abnormal fetal heart rate patterns, and signs of maternal shock. Timely diagnosis through detailed obstetric and past history, vigilant clinical assessment, ultrasound imaging, and continuous fetal monitoring is crucial for mitigating adverse outcomes.

Given its rarity, the management of uterine rupture necessitates a timely and multidisciplinary approach involving obstetricians, anesthetists, and urosurgeons. Modern diagnostic modalities, such as ultrasound and magnetic resonance imaging, are decisive in promptly identifying uterine rupture. Once diagnosed, immediate surgical intervention, often via emergency exploratory laparotomy, with or without uterine repair or obstetric hysterectomy, remains the cornerstone of treatment.

This unique case presentation details the management of uterine rupture in an unscarred multiparous woman, emphasizing the importance of antenatal registration, an early high degree of suspicion, the clinical course, diagnostic challenges, a surgical intervention, and postoperative care.

## Case presentation

A 36-year-old female, who was a gravida of 3, parity of 2 with two previous pregnancies (G3P2L2), presented to the casualty with sudden onset abdominal pain and foul-smelling discharge for two days. She had vaginal deliveries at term for both previous pregnancies that resulted in live births and had no notable medical or surgical histories. There was no mention of a current pregnancy or period of amenorrhea in the presenting complaints. The patient’s history revealed a need for instrumental vaginal delivery in both previous deliveries, where the baby weights were 1.9 and 2.1 kilograms, respectively. No other significant risk factors were present. The casualty medical officer suspected a gynecological ailment for which they gave a referral to our department. On arrival at the emergency room, we quickly noticed that the patient was in an immense amount of pain. On a per abdomen examination, it was noted that the patient had a uterine size corresponding to a full-term pregnancy with fetal parts felt on palpation. The fetal head was 0/5th palpable, with the presence of tenderness and mild bulging in the infraumbilical region. The fetal heart rate was not recordable, prompting urgent intervention.

Diagnosis and decision

The patient was previously unaware of the pregnancy and was neither antenatally registered nor immunized in the current pregnancy. In the absence of any scans or reliable history, we proceeded with a per vaginal examination, which revealed that the cervical os was fully dilated and fully effaced, with the fetal head in occipito-posterior position, absent membranes, a well-formed caput with head at +1 coming to +2 station, and the presence of foul-smelling tobacco juice-like discharge in a borderline pelvis. Mild suprapubic bulging was noted even after emptying the bladder. Additionally, loss of uterine contractions was noted.

When the patient arrived at 1:00 PM, her blood pressure was 140/86 mmHg, and her heart rate was 114 bpm with sinus tachycardia on echocardiogram. Given the clinical scenario, a provisional diagnosis of G3P2L2 with wrong dates and obstructed labor with intrauterine fetal demise with suspected uterine rupture was deduced.

The findings of a diagnostic ultrasound were consistent with intrauterine fetal demise. An emergent cesarean section was done considering the critical maternal state and compromised fetal status, with a high degree of suspicion of a ruptured uterus.

Surgical intervention

**Figure 1:**
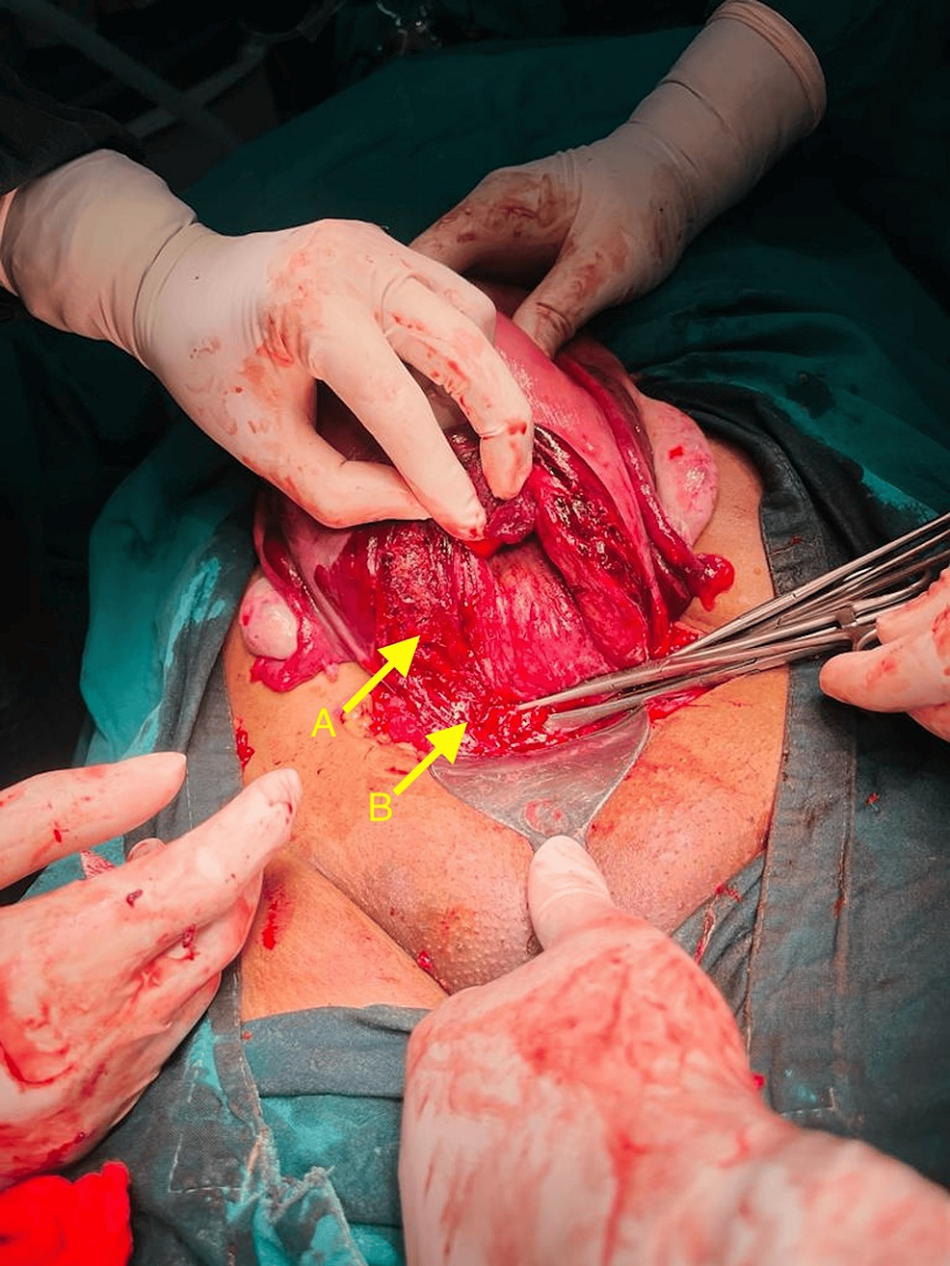
Intraoperative view of uterine rupture. A: uterine rupture B: uterovesical fold dissection

**Figure 2:**
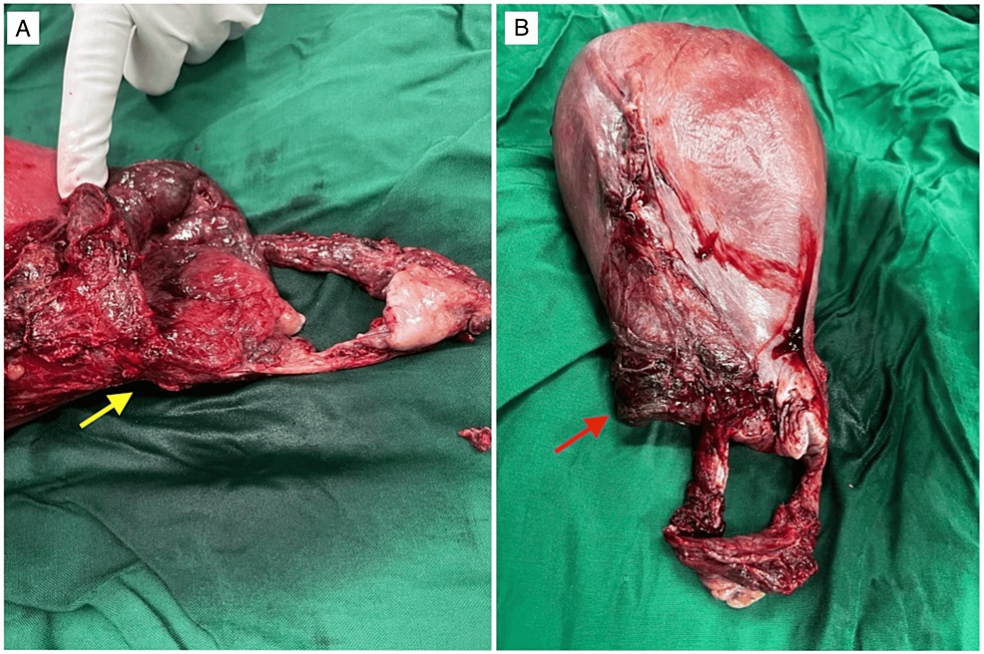
Postoperative specimen showing uterine rupture. A: lateral view Yellow arrow: necrotic edges B: longitudinal view Red arrow: downward and lateral extensions

A multidisciplinary team comprising obstetricians, anesthesiologists, and neonatologists was assembled for the emergent cesarean section. Under general anaesthesia with a Pfannenstiel incision, the surgical approach was an exploration of intrabdominal structures, assessing the uterine condition and delivering the macerated fetus. On entering the abdomen, foul-smelling hemoperitoneum was noted; the uterus was ruptured, with bilateral broad ligament involvement with lateral downward extension and no evidence of placental or structural anomalies, as seen in Figures 1, 2. 

Intraoperatively, a decision to conduct an obstetric hysterectomy was taken after explaining the condition of the patient to the relatives and acquiring consent, owing to the extent of the rupture site. The uterovesical fold was meticulously dissected, and the bladder was pushed downward and reflected. Then the bilateral uterine arteries were clamped, and the downward lateral extension was reassessed and noted to be extended to the vaginal angle before clamping Mackenrodt’s ligament and uterosacral ligament. No ureteric pulsatile movements were noted. Given the above findings and to prevent ureteric injury, emergency cystoscopic ureteric Double-J stenting was carried out by a urosurgeon. Finally, the obstetric hysterectomy was completed. 

Postoperative management

The patient received close monitoring in the intensive care unit, and transfusions of 2 units of packed red blood cells and fresh frozen plasma, each, were given with antibiotic prophylaxis to prevent infection. The maternal prognosis was favorable, and the patient was discharged in stable condition after a thorough postoperative evaluation on postoperative day 19.

The histopathological report of the uterine specimen was suggestive of acute endomyometritis with necrotizing cervicitis, extensive hemorrhage, and ischemic changes.

## Discussion

The rupture of an unscarred uterus is undeniably a major, life-threatening emergent condition, defined as a complete or partial tear of the uterine wall in a pregnant uterus, often involving the myometrium and the overlying serosa. It can present with per vaginal bleeding, acute abdomen and fetal distress, and atypical contractions. Most uterine ruptures occur in women with a history of previous cesarean sections or other uterine surgeries, so unscarred uterine ruptures present a unique challenge due to their atypical clinical presentation, rarity, lack of timely diagnosis, and potential for severe consequences.

The overall incidence of uterine rupture was around 0.2 per 10,000 women [5], and rupture in an unscarred uterus was observed at a rate of 2 in 39,529, approximately 0.0051% [6]. By contrast, uterine rupture occurred in 0.8% of scarred uteri [7]. However, the incidence of uterine rupture varies globally, so the exact incidence is difficult to determine [8].

Occurrence of unscarred uterine rupture is associated with factors such as advanced maternal age of over 35 years old, multiparity, having a postdated pregnancy, multifetal gestation, the utilization of labor induction or augmentation, traumatic history (e.g., manual or instrumental obstetric maneuvers), external traumas, domestic violence, manual placental removal, and the presence of infective etiologies (e.g., chorioamnionitis) [8-11]. Rupture of the uterus is more commonly observed in women attempting vaginal birth after a cesarean or hysterotomy. It is also more common in women with a previous history of fetal surgery or uterine surgery, such as myomectomy or septoplasty. Scarred uteri are most vulnerable to rupture in the late third trimester of pregnancy or during labor [12]. 

Most unscarred uterine ruptures do not have notable risk factors, and it is important to note that this is an exceptionally rare event. In the absence of any known causative factor, it may be wise to presume a concealed history of previous pregnancy or surgery [8]. In addition, we must consider factors such as an unreliable history of unwanted pregnancy or unsafe abortions in the context of social taboos and cultural practices in developing countries.

Our case is a multigravida female with a previously unscarred uterus of advanced maternal age who was antenatally unbooked and reported with intrauterine fetal demise. Though there is no notable cause, possible risk factors like advanced maternal age, multigravida, prolonged labour, and previous history of operative vaginal delivery might have possibly contributed to the uterine rupture in this case. In such a situation, we prioritized maternal well-being and condition. Clinical acumen and a high degree of suspicion are paramount to prevent the loss of golden time. The use of resources such as ultrasound and magnetic resonance imaging is of added value whenever available and practical. Mourad et al. reported a similar case of a rupture in an unscarred uterus; the authors had opined that premature rupture of membranes with an abruption of placenta with Couvelaire uterus could be the most likely event in pathophysiology; however, unlike our case, a hysterectomy was not performed because the rupture and damage were to a lesser extent [13].

Sakr et al. reported a similar case with a scarred uterus [3], and Purdie et al. reported a ruptured uterus with disseminated intravascular coagulation [4]. The occurrence of uterine rupture poses life-threatening complications for both the mother and the infant. Primary indicators typically involve intense abdominal pain, frequently coupled with fetal distress, atypical heart rate patterns, and maternal shock [9]. It is imperative to promptly diagnose and intervene to minimize the risks linked with uterine rupture.

Uterine rupture, a critical obstetric emergency, typically requires immediate surgical intervention, commonly via emergency cesarean section, aiming to promptly deliver the baby and repair the uterine tear [12]. Extensive ruptures may necessitate a hysterectomy to control bleeding and avert further complications. Healthcare providers should prioritize preventing complications and ensuring successful patient outcomes, such as ureteric DJ stenting [14]. A multidisciplinary approach involving urologists, anesthesiologists, and intensivists can significantly mitigate complications, as observed in the current case [14]. Preventing uterine rupture in unscarred uteri involves thorough antenatal care, vigilant labor monitoring, and elective cesarean delivery in some cases. Monitoring for coagulation factors and signs of shock is crucial, with supportive measures such as intravenous fluids and blood transfusions aiding stabilization in cases of significant blood loss [12]. Management decisions hinge on the severity of the rupture and the maternal-fetal condition.

## Conclusions

Rupture in an unscarred uterus, though rare, poses a significant threat to both maternal and fetal well-being, necessitating a judicious balance between prompt identification and coordinated medical interventions. Our case and others similar to this, push the importance of thorough antenatal care, vigilant labor monitoring, a multidisciplinary approach, clinical suspicion, timely intervention, and anticipatory measures in high-risk pregnancies. Although the risk factors for unscarred uterine rupture are not entirely preventable, continuous research and shared clinical experiences are imperative to refine management strategies and optimize outcomes. Addressing psychological impacts and providing postpartum support is also a crucial part of the management of such patients.

Therefore, synthesizing current knowledge from scientific literature can enhance the understanding of such cases in diverse obstetric settings. Ultimately, uterine rupture poses a significant challenge to the mother, child, and attending obstetrician, underscoring the importance of maintaining a delicate equilibrium among this trio for favorable results.
